# MiR-195-5p regulates oxidative stress and aerobic metabolism by directly downregulating *GLS2* in high glucose-induced human lens epithelial cells

**DOI:** 10.20945/2359-4292-2024-0469

**Published:** 2025-08-04

**Authors:** Ling Yao, Meng Yue, Yuxian Sun, Juan Li, Qi Zhou, Ning Li, Xiaoli Yue, Junyan Hu, Linkang Yin, Zhengyang Xu, Xiang Gao, Wei Zhang, Ziqing Gao

**Affiliations:** 1 Department of Ophthalmology, The First Affiliated Hospital of Bengbu Medical University, Bengbu City 233004, Anhui Province, China; 2 Department of Ophthalmology, Tianjin Eye Hospital, Tianjin 300020, China; 3 Department of Ophthalmology, Tianjin Key Lab of Ophthalmology and Visual Science, Tianjin 300020, China; 4 Department of Ophthalmology, Clinical College of Ophthalmology Tianjin Medical University, Tianjin 300020, China

**Keywords:** MicroRNAs, Glutaminase, Lens Epithelial Cells, Cataract, Oxidative stress

## Abstract

**Objective:**

To investigated how miR-195-5p affects oxidative stress and modulates aerobic metabolism.

**Materials and methods:**

MiR-195-5p plus *GLS2* mRNA was identified by conducting real-time quantitative polymerase chain reaction. Western blotting was conducted to determine *GLS2* protein expression. Corresponding kits were used to determine the concentrations of glutamate, reduced glutathione, oxidized glutathione, a-ketoglutarate, and adenosine triphosphate. The cell counting Kit-8 assay was performed to determine viability. Flow cytometry assay was performed to measure the reactive oxygen species content. Finally, a dual-luciferase reporter assay was conducted to confirm the interaction of miR-195-5p with *GLS2* mRNA in the 3’UTR.

**Results:**

In high glucose-induced SRA01/04 cells, miR-195-5p was overexpressed, and *GLS2* was downregulated. When miR-195-5p was upregulated, the levels of glutamate, reduced glutathione, a-ketoglutarate, and adenosine triphosphate, along with the reduced glutathione-to-oxidized glutathione ratio decreased, whereas the reactive oxygen species levels increased. Oxidative stress was ameliorated after miR-195-5p was downregulated. MiR-195-5p adversely controls the expression of *GLS2* mRNA and protein. MiR-195-5p exacerbates oxidative damage and hinders aerobic metabolism by downregulating *GLS2*.

**Conclusion:**

Oxidative stress and aerobic metabolism in human lens epithelial cells were found to be regulated by miR-195-5p after the downregulation of *GLS2*.

## INTRODUCTION

Diabetes mellitus (DM) is characterized by chronic multisystem dysfunction and results in ocular complications, such as diabetic cataracts (DCs), which are among the primary late complications of DM (^1^). Cataractogenesis is associated with three molecular mechanisms, including nonenzymatic glycation, oxidative stress, and the polyol pathway in blood glucose conversion (^2^). In this study, the effect of oxidative stress on DCs was comprehensively investigated.

Glutaminase catalyzes the hydrolysis of glutamine into glutamate in mitochondria. The glutaminase family includes two genes in humans (*GLS* and *GLS2*) on chromosomes 2 and 12, respectively (^3^). Although glutaminases are mitochondrial enzymes, *GLS2* is located in the cell nucleus, indicating that *GLS2* has a direct role or is a coregulator in the final transcriptional generation of glutamine (^4^). *GLS2* comprises an ankyrin-repeat domain in the carboxyl terminus, has different characteristics, and performs various functions via protein-protein interactions. *GLS2* generates reduced glutathione (GSH) and nicotinamide adenine dinucleotide (NADH) to acquire antioxidant ability and reduce intracellular reactive oxygen species (ROS) concentrations. The interconversion between oxidized glutathione (GSSG) and GSH can counteract the damaging effects of oxidants on sulfhydryl (-SH) groups, protecting sulfhydryl-containing proteins and enzymes from oxidation and denaturation when the balance between GSSG and GSH is disrupted (^5^). Reactive oxygen species and free radicals can directly attack sensitive groups such as sulfhydryl (-SH) groups in lens proteins, leading to structural disruption and aggregation of the proteins (^6^). Therefore, maintaining normal levels of GSSG (as well as GSH) plays a key role in preserving the transparency of the lens.

MicroRNAs (miRNAs) are found in various ocular tissues, such as the lens and retina, and play a role in multiple ocular disorders (^7^). Additionally, miRNAs are specifically expressed in transparent and cataractous lenses, indicating that these miRNAs play key roles in cataract formation (^8^). Located on chromosome 17p13.1, the miR-195 gene generates miR-195-3p plus miR-195-5p. The latter is a member of the family of miR-15a/b/16/195/497, which has different functions in various diseases (^9^). For example, in the case of breast cancer, miR-195-5p targets Cyclin E1 as a tumor promoter (^10^). Additionally, miR-195-5p modulates vascular endothelial growth factor A to inhibit acute kidney injury (^11^). Zhang and cols. (^12^) reported that oxidative stress upregulated miR-195 in the retina of diabetic rats and also in cultured retinal endothelial cells exposed to high glucose (HG). They also found that miR-195 accelerated oxidative stress-induced retinal endothelial cell injury by targeting mitofusin 2 in diabetic rats. Purohit and cols. (^13^) reported that miR-195 impaired mitochondrial function and dynamics by targeting mitofusin-2 in breast cancer cells, concomitantly increasing SOD-2 to maintain moderate levels of oxidative stress. Moreover, silencing miR-195 reduced diabetic cardiomyopathy in a mouse model of streptozotocin-induced type 1 diabetes, at least partially by reducing oxidative stress damage via B-cell leukemia/lymphoma 2 and sirtuin 1 (^14^). Another study found that HG induces metabolic memory in the retinal pigment epithelium through oxidative stress and mitochondrial damage by regulating miR-195 and bcl-2 (^15^).

However, the underlying mode of action of miR-195-5p in HG-induced human lens epithelial cells (HLECs) should be determined. Hence, this study was conducted *in vitro* to examine how miR-195-5p affects oxidative stress and aerobic metabolism. The objective of this study was to investigated how miR-195-5p affects oxidative stress and modulates aerobic metabolism.

## MATERIALS AND METHODS

### Cell culture plus disposal

The HLECs (SRA01/04) were purchased from LMAI Bio (Shanghai, China), incubated in a culture medium consisting of 1% penicillin-streptomycin (10,000 U/mL; Gibco, Carlsbad, CA, USA) 15140-122 mixed with FBS (10%; Gibco)10099-141, and stored at 37°C with 5% CO_2_. Human lens epithelial cells treated with 25 mM glucose (high dose) were used as the experimental group, whereas those treated with 5 mM glucose (low dose) were used as the control group for 24 hours. (^16^)

### Cell transfection

The following reagents from Sangon Biotech (Shanghai, China) were used: miR-195-5p mimics along with negative control (NC) (miR-NC); siRNAs targeting GLS2 mRNA (si-GLS2#1 and si-GLS2#2) plus a paired control (si-NC); and miR-195-5p inhibitors plus the NC (anti-miR-NC). To construct the knockdown or overexpression vector, GLS2 siRNA, NC siRNA, and GLS2 fragments were cloned and inserted into pcDNA3.1 (Invitrogen, Carlsbad, CA, USA), and the cells were transfected for 12 hours at 37°C with a Lipofectamine™ 3000 kit (Invitrogen, 11668019) following the manufacturer’s instructions. The target sequences of GLS2 siRNA (RNAi 1) were 5’-GGACACATCGAAGTTGTTAAA-3’ and (RNAi 2) 5’-ATC AAGATGGACTGTAACAAA-3’. The 5’- ACTAAGACTACTGTTCCAA -3’ sequence was used as the NC siRNA. All transfection experiments were conducted on the aforementioned cellular model treated with HG. The experiment was conducted independently three times for statistical analysis.

### Cell viability assay

SRA01/04 cells (5´10^3^/well) were maintained in 96-well plates for 24 hours and then treated with CCK-8 solution (10 µL/well; MeilunBio^®^, Dalian, China) and WST-8 at 37°C. After 2 hours, a microplate reader (Thermo Fisher Scientific, Waltham, MA, USA) was used to detect the solubility at 450 nm. The experiment was conducted independently three times for statistical analysis.

### Real-time quantitative polymerase chain reaction assay

TRIzol reagent (Life Technologies, Carlsbad, California, USA) 15596018 was used to extract total RNA, and the kit for reverse transcription into cDNA was purchased from Thermo Fisher Scientific (Shanghai, China) #K1622. Similarly, RT Master Mix (TaKaRa; PrimeScript, Dalian, China) RR036A was used to reverse-transcribe *GLS2* mRNA into cDNA. Additionally, SYBR® Green Master Mix (Universal, FastStart; Roche, Shanghai, China) 04 913 914 001 was used to perform real-time quantitative polymerase chain reaction (qRT-PCR). To standardize the expression of miR-195-5p, RNA U6 and glyceraldehyde phosphate dehydrogenase (GAPDH) were used for standardizing *GLS2* mRNA. The data were analyzed using the 2^-△△Ct^ method. The designed primers are listed in **Table 1**. The experiment was conducted independently three times for statistical analysis.

**Table 1:** Designed primers table

Gene	Sequence (5′→3′)
miR-195-5p	F: 5’-AGGGTAGCAGCACAGAAAT-3’ R: 5’-GTGCAGGGTCCGAGGT-3’
GLS2 mRNA	F: 5’-TCTCTTCCGAAAGTGTGTGAGC-3’ R: 5’-CCGTGAACTCCTCAAAATCAGG-3’
RNA U6	F: 5’-GCAAATTCGTGAAGCGTT-3’ R: 5’-GTGCAGGGTCCGAGGT-3’
GAPDH	F: 5’-GAGAAGGCTGGGGCTCATTT-3’ R: 5’-TAAGCAGTTGGTGGTGCAGG-3’

### Western blotting

RIPA lysis buffer (Beyotime, Shanghai, China) containing P0013B and a Western blotting assay kit (Nanjing Jiancheng, Nanjing, China) containing W001-1-2 were used to assemble the total protein from SRA01/04 cells following the manufacturer’s instructions. After boiling with a buffer solution, different proteins isolated via SDS-PAGE were transferred onto PVDF membranes (Millipore, Billerica, MA, USA) (IPVH00010) and incubated overnight with primary antibodies (4 °C). Then, a secondary antibody to which horseradish peroxidase was added was used to hatch the membranes. The results were visualized with chemiluminescence using an ECL kit (Millipore);(WBKLS0100). The primary and secondary antibodies used were as follows: anti-GLS2 (1:2000);(Abcam, ab113509), anti-GAPDH (1:5000;(Abcam, ab8245), HRP-conjugated goat anti-mouse (1:50000);(Abcam, ab205719), and HRP-conjugated goat anti-rabbit (1:50000);(Abcam, Ab6721). The experiment was conducted independently three times for statistical analysis.

### Reactive oxygen species assay

Reactive oxygen species levels were determined using a DCFH-DA ROS assay kit (Abcam, ab113851) following the manufacturer’s instructions. After transfection, the SRA01/04 cells were incubated with HG for 24 hours. The resuspended cells (1´10^5^) in a buffer solution containing DCFH-DA were subsequently cultivated at 37°C for 15 minutes in a dark chamber and analyzed using a flow cytometer after 1 hour. The experiment was conducted independently three times for statistical analysis.

### Detection of indicators of oxidative stress

The cell pellets were collected from each treatment group, an appropriate amount of isotonic phosphate-buffered saline (PBS) was added to grind and disrupt the cells, the mixture was centrifuged at 2,500 rpm for 10 minutes, and the supernatant was removed to determine the protein concentration; 0.2 mL of supernatant was added, 0.6 mL of reagent was added, the mixture was mixed thoroughly at 3,000 to 3,500 rpm, the mixture was centrifuged for 10 minute, 0.5 mL of supernatant was collected, the test was performed following the instructions provided with the kit, the reagent was added according to the instructions, and the absorbance was measured at the wavelength recommended by the manufacturer. Kits for detecting a-ketoglutarate (H465), adenosine triphosphate (ATP); (A095-1-1), glutamate (A074-1-1), GSH (A006-2-1), and GSSG (A061-1-2) were purchased from Nanjing Jiancheng Company. The experiment was conducted independently three times for statistical analysis.

### Dual-luciferase assay

TargetScanHuman 8.0 (available online) was used to predict the targets of miR-195-5p and the targets of *GLS2* mRNA. Lipofectamine™ 3000 reagent was used for cloning the pmirGLO vector (Life Technologies, Carlsbad, CA, USA), which was cotransfected with pGL3-*GLS2*-wild-type (WT) and pGL3-*GLS2*-mutant (MUT1) sequences, thereby facilitating the construction of luciferase plasmids. Subsequently, SRA01/04 cells were cotransfected with the luciferase plasmid and either miR-NC or miR-195-5p. At 24 hours post-transfection, luciferase activity was assessed using the Dual-Luciferase® Reporter Assay (Promega, Madison, WI, USA) following the manufacturer’s instructions. The experiments were performed in triplicate for statistical analysis.

### Statistical analysis

All data were analyzed using Statistical Package for the Social Sciences (SPSS), version 25.0 (IBM, Chicago, IL, USA). Normally distributed data were used for data analysis, and the data are presented as the mean ± standard deviation (SD). Every experiment was conducted independently three times. The differences between groups were determined by conducting paired Student’s *t*-test, whereas those among multiple groups were determined by conducting one-way analysis of variance (Anova). *Post hoc* multiple comparisons were made by conducting the SNK-Q test. All differences were considered to be statistically significant at p < 0.05 (two-tailed).

## RESULTS

### MiR-195-5p and *GLS2* mRNA and protein levels in high glucose-induced human lens epithelial cells

**Figure 1:**
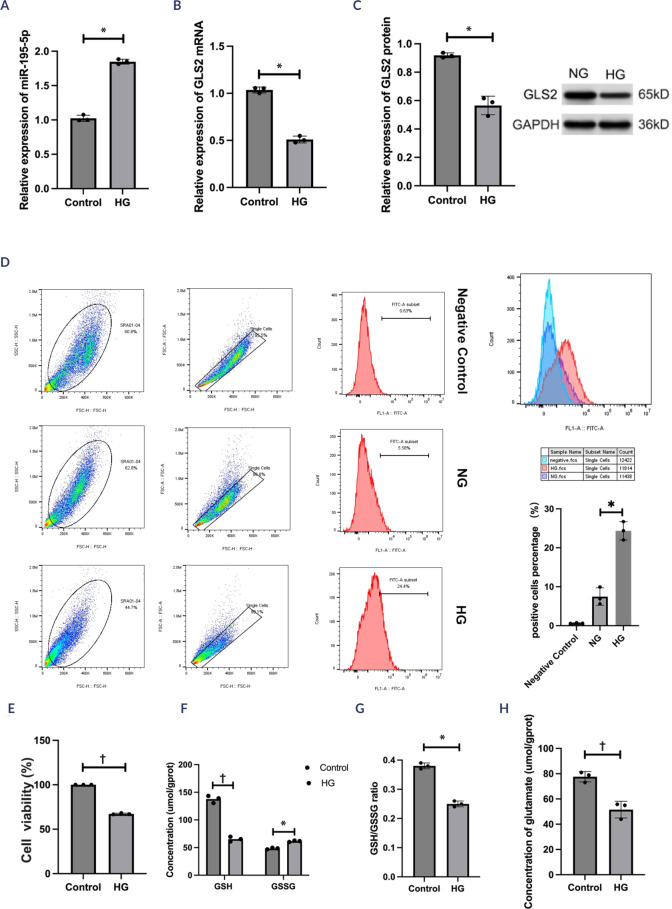
MiR-195-5p and *GLS2* mRNA and protein levels in high glucose-treated human lens epithelial cells. (**A**) The expression of miR-195-5p was determined by real-time quantitative polymerase chain reaction. (**B**) *GLS2* mRNA expression was determined by real-time quantitative polymerase chain reaction. (**C**) Western blotting analysis was conducted to detect *GLS2* protein expression. (**D**) Flow cytometry was performed to determine reactive oxygen species levels. (**E**) Cell viability was determined using a CCK-8 kit. (F-H) Glutamate, reduced glutathione, and oxidized glutathione levels were measured using corresponding kits. The experiment was conducted independently three times for statistical analysis. * p < 0.05; † p < 0.01. HG: high glucose; NG: normal glucose; GSH: reduced glutathione; GSSG: oxidized glutathione

Higher miR-195-5p expression was detected in the experimental group than in the normal control group (t = -18.07; p < 0.05; **Figure 1A**). In contrast, lower *GLS2* mRNA (t = 13.78; p < 0.05; **Figure 1B**) and *GLS2* protein (t = -18.07; p < 0.05; **Figure 1C**) expression was found in HG-induced lens cells than in normal lens cells. The ROS levels in HG-induced lens cells were higher than those in normal lens cells (t = -14.68; p < 0.05; **Figure 1D**). The results of the CCK-8 assay suggested that cell viability was lower in HG-induced HLECs than in NG-induced HLECs (t = 72.96; p < 0.01; **Figure 1E**). Similarly, the GSH level (t = 11.47; p < 0.05; **Figure 1F**), GSH-to-GSSG ratio (t = 9.00; p < 0.05; **Figure 1G**), and glutamate level (t = 52.27; p < 0.01; **Figure 1H**) were lower in HG-treated HLECs than in NG-treated HLECs. These findings suggested that elevated miR-195-5p levels and reduced *GLS2* mRNA and protein expression are associated with hyperglycemia and oxidative stress.

### Role of miR-195-5p in influencing oxidative stress and aerobic metabolism *in vitro*

**Figure 2:**
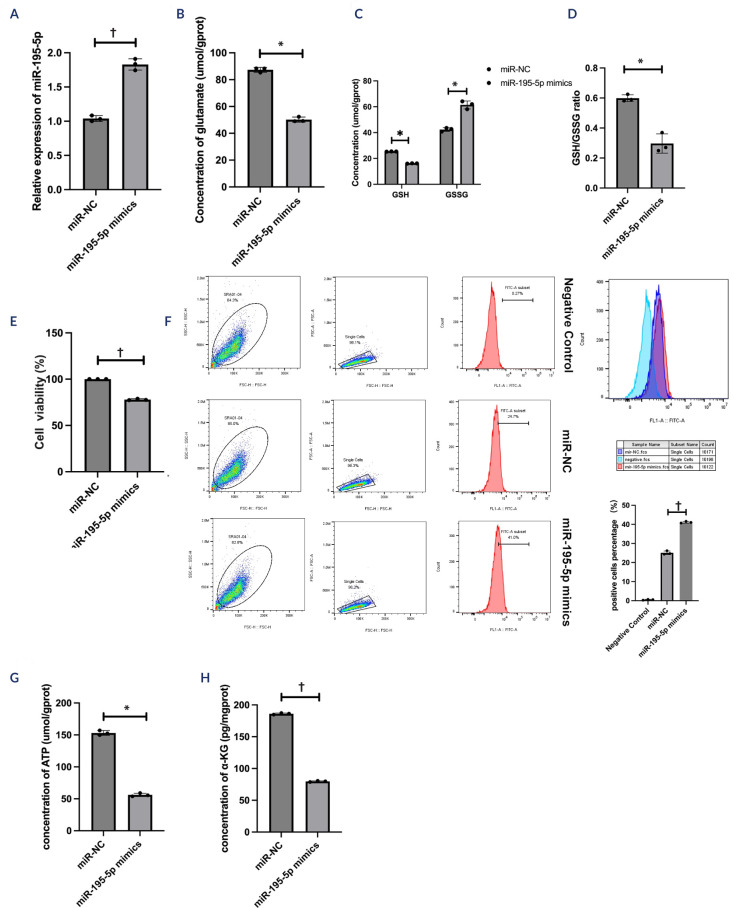
The role of miR-195-5p mimics in oxidative stress and aerobic metabolism in high glucose-treated human lens epithelial cells. (**A**) The relative expression of miR-195-5p was determined in SRA01/04 cells after miR-negative control and miR-195-5p mimic transfection. (**B-D**) The levels of glutamate, reduced glutathione, and oxidized glutathione were determined using corresponding kits. (**E**) Cell viability was determined using a CCK-8 assay kit. (**F**) Flow cytometry was performed to determine the reactive oxygen species levels. (**G-H**) Corresponding kits were used to measure adenosine triphosphate and a-ketoglutarate levels. The experiments were conducted independently three times for statistical analysis. * p < 0.05; † p < 0.01. NC: negative control; GSH: reduced glutathione; GSSG: oxidized glutathione.

**Figure 3:**
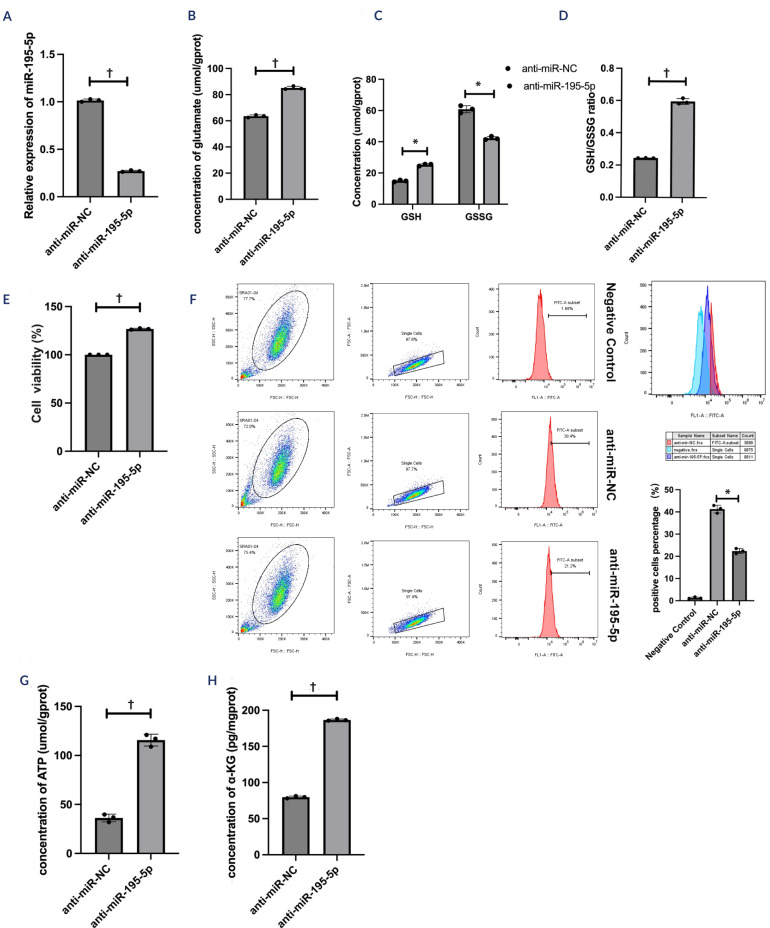
The role of miR-195-5p inhibitors in oxidative stress and aerobic metabolism in high glucose-treated human lens epithelial cells. (**A**) The relative expression of miR-195-5p was determined in SRA01/04 cells after anti-miR-negative control and anti-miR-195-5p transfection. (**B-D**) The levels of glutamate, reduced glutathione, and oxidized glutathione were determined using corresponding kits. (**E**) Cell viability was determined using a CCK-8 assay kit. (**F**) Flow cytometry was performed to detect the reactive oxygen species levels. (**G-H**) adenosine triphosphate and a-ketoglutarate levels were measured using corresponding kits. The experiments were conducted independently three times for statistical analysis. * p < 0.05; † p < 0.01. NC: negative control; GSH: reduced glutathione; GSSG: oxidized glutathione.

SRA01/04 cells were transfected with miR-NC, miR-195-5p mimics, anti-miR-195-5p, or anti-miR-NC for 48 h. The efficacy of the miR-195-5p mimics (t = -11.88; p < 0.01; **Figure 2A**) or inhibitors (t = 58.19; p < 0.01; **Figure 3A**) was validated via qRT-PCR. The contents of glutamate (t = 34.73; p < 0.05; **Figure 2B**), GSH (t = 240.97; p < 0.05; **Figure 2C**), and the GSH-to-GSSG ratio (t = 5.19; p < 0.05; **Figure 2D**) were significantly lowered by the miR-195-5p mimics, whereas the contents of glutamate (t = -17.79; p < 0.01; **Figure 3B**), GSH (t = -20.71; p < 0.05; **Figure 3C**), and the GSH/GSSG ratio (t = -33.63; p < 0.01; **Figure 3D**) were significantly elevated by the miR-195-5 inhibitors. Additionally, the decrease in cell viability was attributed to the miR-195-5p mimics (t = 45.76; p < 0.01; **Figure 2E**), whereas the inhibitors increased cell viability (t = -64.05; p < 0.01; **Figure 3E**). The results of the flow cytometry assays revealed that the ROS levels were increased via the miR-195-5p mimics (t = -47.49; p < 0.01; **Figure 2F**), whereas the inhibitors decreased the ROS levels (t = 29.78; p < 0.05; **Figure 3F**). Moreover, ATP (t = 30.19; p < 0.05; **Figure 2G**) and a-ketoglutarate (t = 383.00; p < 0.01; **Figure 2H**) levels decreased when the miR-195-5p mimics were used, whereas the inhibitors increased the levels of ATP (t = -64.64; p < 0.01; **Figure 3G**) and a-ketoglutarate (t = -385.00; p < 0.01; **Figure 3H**). These findings indicated that miR-195-5p exacerbated oxidative damage and affected aerobic metabolism.

### *GLS2* mRNA and protein were negatively regulated by miR-195-5p

**Figure 4:**
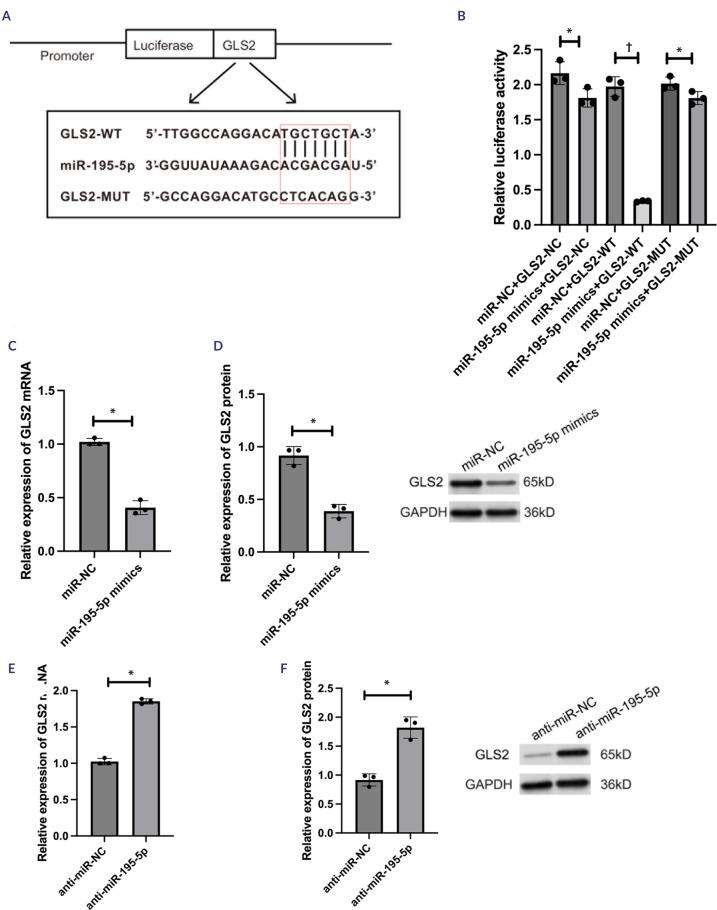
A negative correlation was found between the expression products of *GLS2* and miR-195-5p. (**A**) The binding sites of miR-195-5p predicted at the *GLS2* 3’UTR or the mutational 3’UTR of *GLS2* mRNA were verified. (**B**) SRA01/04 cells were transfected with miR-NC, miR-195-5p mimics, WT-*GLS2*, or MUT-*GLS2*, and the luciferase reporter activities of each group were measured. (**C**) Comparison between the miR-NC and miR-195-5p mimic groups. *GLS2* mRNA expression was determined by real-time quantitative polymerase chain reaction. (**D**) Comparison between the miR-NC and miR-195-5p mimic groups. *GLS2* protein expression was determined by Western blotting assays. (**E**) Comparison between the anti-miR-NC and miR-195-5p inhibitor groups. *GLS2* mRNA expression was identified by real-time quantitative polymerase chain reaction. (**F**) Comparison of the anti-miR-NC and miR-195-5p inhibitor groups. *GLS2* protein expression was measured by conducting Western blotting assays. The experiments were conducted independently three times for statistical analysis. * p < 0.05; † p < 0.01.

The probable candidates were identified via bioinformatic prediction to elucidate the underlying mechanism by which miR-195-5p affects oxidative damage. The binding site sequence of miR-195-5p was subsequently used to construct MUT1-*GLS2* plus WT-*GLS2* plasmids (**Figure 4A**). The activity of the reporter was inhibited following miR-195-5p mimic and WT-*GLS2* cotransfection (*F* = 210.48; p < 0.01; **Figure 4B**). In contrast to the effects of miR-NC, the miR-195-5p mimics attenuated *GLS2* mRNA (*t* = 13.16; p < 0.05; **Figure 4C**) and protein (*t* = 36.74; p < 0.05; **Figure 4D**) expression. Moreover, anti-miR-NC plus anti-miR-195-5p was transfected into HG-treated HLECs. The *GLS2* mRNA (*t* = -18.24; p < 0.05; **Figure 4E**) and protein (*t* = -5.89; p < 0.05; **Figure 4F**) levels were significantly higher in the miR-195-5p inhibitor group than in the anti-miR-NC group. These findings suggested that the *GLS2* expression product was negatively associated with miR-195-5p. Additionally, miR-195-5p targeted the *GLS2* gene.

### Regulation of oxidative stress and aerobic metabolism by miR-195-5p depends on *GLS2*

**Figure 5:**
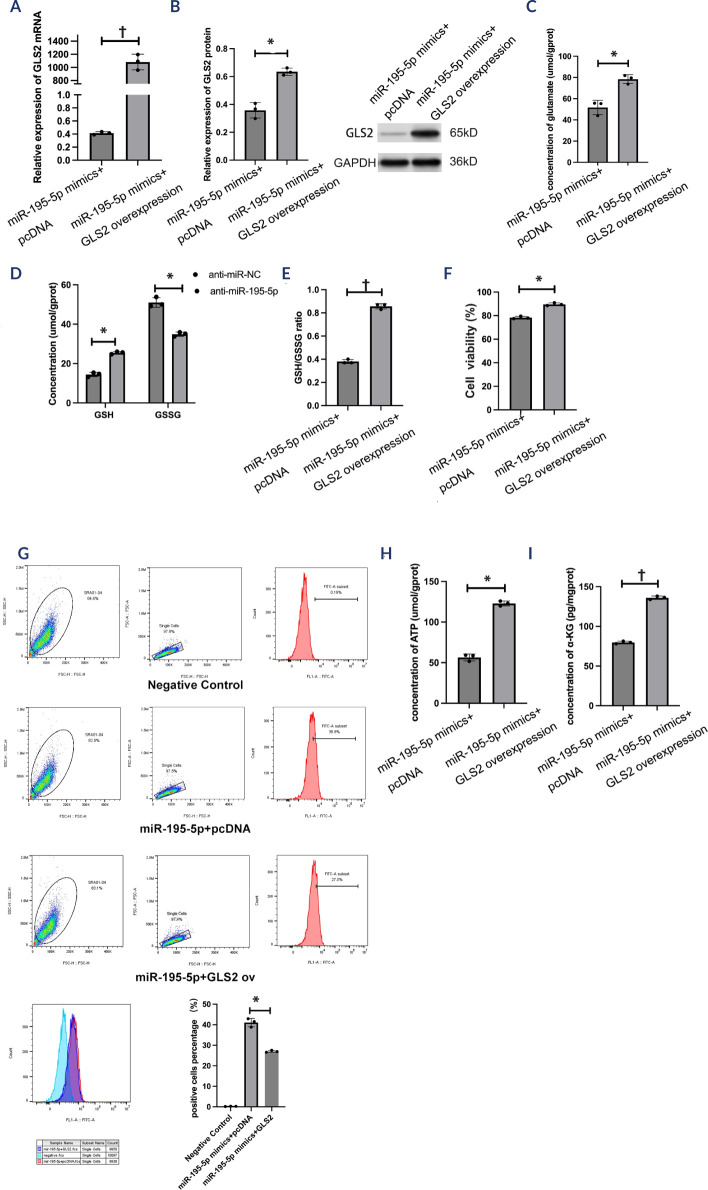
MiR-195-5p mimics mediate oxidative stress and aerobic metabolism in a *GLS2*-dependent manner. (**A**) *GLS2* mRNA expression was determined by real-time quantitative polymerase chain reaction. (**B**) *GLS2* protein expression was identified by Western blotting analysis. (**C-E**) The levels of glutamate, reduced glutathione, and oxidized glutathione were determined using corresponding kits. (**F**) Cell viability was determined using a CCK-8 assay kit. (**G**) Flow cytometry was performed to determine the reactive oxygen species levels. (**H-I**) Adenosine triphosphate and a-ketoglutarate levels were tested using corresponding kits. The experiments were conducted independently three times for statistical analysis. * p < 0.05; † p < 0.01.

**Figure 6:**
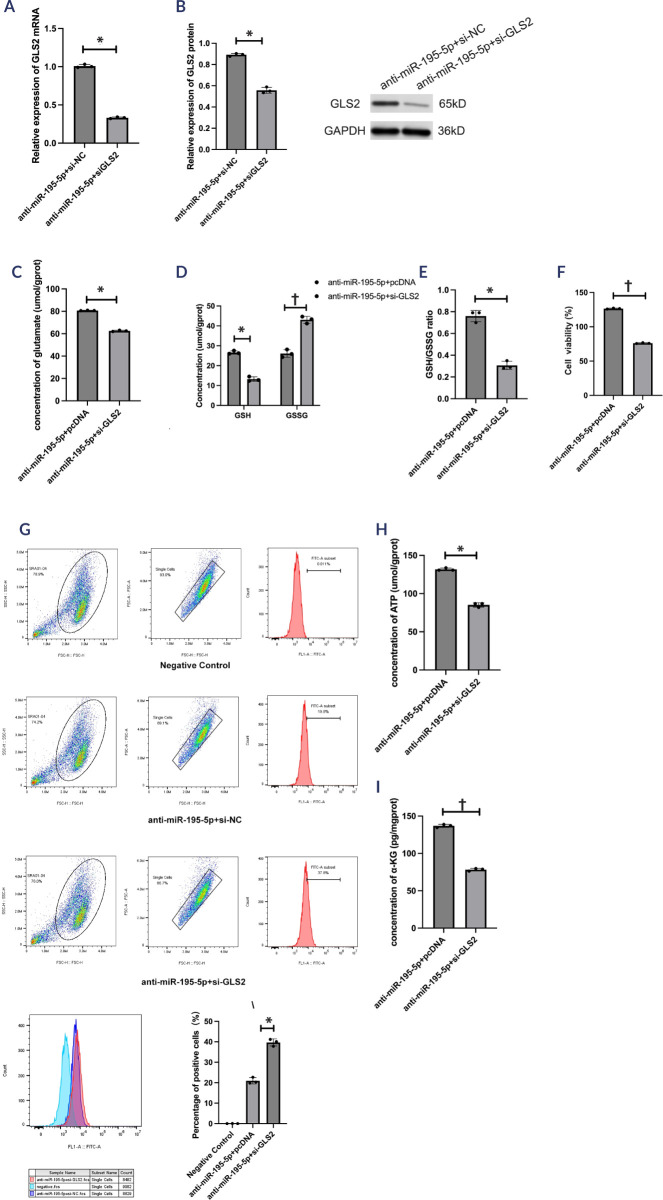
MiR-195-5p inhibitors mediate oxidative stress and aerobic metabolism via *GLS2*. (**A**) *GLS2* mRNA expression was determined by real-time quantitative polymerase chain reaction. (**B**) *GLS2* protein expression was determined by Western blotting. (**C-E**) The levels of glutamate, reduced glutathione, and oxidized glutathione were determined using corresponding kits. (**F**) Cell viability was determined using a CCK-8 assay kit. (**G**) Flow cytometry analysis was conducted to determine the levels of reactive oxygen species. (**H-I)** Corresponding kits were used to measure the levels of adenosine triphosphate and a-ketoglutarate. The experiments were conducted independently three times for statistical analysis. * p < 0.05; † p < 0.01.

To determine whether the effect of miR-195-5p on oxidative stress and aerobic metabolism is mediated by *GLS2*, miR-195-5p mimics+pcDNA3, miR-195-5p mimics+overexpression (miR-195-5p +*GLS2* ov) of *GLS2*, anti-miR-195-5p+si-NC, and anti-miR-195-5p+si-*GLS2* were transfected into HG-treated HLECs. The former group (*t* = -15.68; p < 0.01; **Figure 5A**; *t* = -10.18; p < 0.05; **Figure 5B**) and the latter group (*t* = 45.43; p < 0.05; **Figure 6A**; *t* = 22.98; p < 0.05; **Figure 6B**) were validated for efficacy by conducting the qRT-PCR and WB assays, respectively. The miR-195-5p +*GLS2* overexpression group was detected with higher levels of glutamate (*t* = -16.25; p < 0.05; **Figure 5C**), GSH (*t* = -64.28; p < 0.01; **Figure 5D**), and the GSH-to-GSSG ratio (*t* = -5.29; p < 0.05; **Figure 5E**), greater cell viability (*t* = -29.01; p < 0.05; **Figure 5F**), and lower levels of ROS (*t* = 11.44; p < 0.05; **Figure 5G**), whereas higher levels of ATP (*t* = -65.85; p < 0.01; **Figure 5H**) and a-ketoglutarate (*t* = -30.96; p < 0.05; **Figure 5I**) were detected in the miR-195-5p mimics+pcDNA3 group. Additionally, lower levels of glutamate (*t* = 40.47; p < 0.05; **Figure 6C**), GSH (*t* = 10.77; p < 0.05; **Figure 6D**), and the GSH-to-GSSG ratio (*t* = 11.99; p < 0.05; **Figure 6E)**, lower cell viability (*t* = 521.03; p < 0.01; **Figure 6F**), higher levels of ROS (*t* = -280.59; p < 0.05; **Figure 6G**), and lower levels of ATP (*t* = -32.57; p < 0.05; **Figure 6H**) and a-ketoglutarate (*t* = 211.00; p < 0.01; **Figure 6I**) were found in the anti-miR-195-5p+si-*GLS2* group compared to that in the anti-miR-195-5p+si-NC group. The results of the phenotypic rescue experiment indicated that *GLS2* overexpression partially counteracted the ability of the miR-195-5p mimics to affect oxidative stress and aerobic metabolism, and vice versa.

## DISCUSSION

The role of miR-195-5p in oxidative stress in HLECs was investigated. First, differences in miR-195-5p expression between HG-treated HLECs and NG-treated HLECs were observed. Specifically, miR-195-5p was upregulated in HG-treated SRA01/04 cells compared to NG-treated SRA01/04 cells. Second, the upregulation of miR-195-5p aggravated oxidative damage, whereas the downregulation of miR-195-5p alleviated oxidative impairment. Third, miR-195-5p was found to directly target *GLS2*. Additionally, miR-195-5p negatively modulated *GLS2*. Fourth, miR-195-5p regulated oxidative stress in a *GLS2*-dependent manner, which was confirmed by a rescue experiment. Moreover, miR-195-5p was found to regulate cellular energy homeostasis by affecting ATP and α-ketoglutarate levels, both of which are important intermediates in mitochondrial oxidative phosphorylation and the TCA cycle, suggesting an influence on aerobic metabolism. However, the term “aerobic metabolism” covers a broader scope, including parameters such as the oxygen consumption rate (OCR) and mitochondrial respiration, which were not directly evaluated in this study. To fully understand the extent of the effect of miR-195-5p on mitochondrial function, future studies should incorporate these measurements systematically.

A study reported the importance of hyperglycemia in the development of DC (^17^). In the present study, 25 mM HG was used to induce SRA01/04 cell lines to simulate an HG environment and determine the effect of HG on cell activity and oxidative stress. The findings suggested that 25 mM HG effectively restrained cell activity and aggravated oxidative damage, along with elevated levels of ROS, suggesting that HG-treated HLECs were created in triplicate. Moreover, the viability of HLECs was considerably decreased by HG, simulating the physiological conditions that occur in the lenses of individuals with diabetes.

Oxidative stress is triggered by an imbalance between oxidant generation and insufficient antioxidative defense, which is considered to be a crucial mechanism in the pathogenesis of DC (^18^). Our cell viability assays revealed that ATP production decreased significantly in the context of miR-195-5p overexpression, indicating compromised metabolic function rather than an increase in ATP production due to apoptosis. These findings support the notion that miR-195-5p affects mitochondrial bioenergetics by regulating *GLS2*. The levels of oxidative damage markers, such as glutamate and GSH, and the GSH/GSSG ratio decreased considerably after the disposal of HG, which agreed with the hypothesis that oxidative stress is associated with DC damage. We found that miR-195 influences oxidative stress through pathways such as SIRT1 and MFN2 in various cell models, including human retinal endothelial cells (HRECs), human microvascular endothelial cells (HMECs), and human umbilical vein endothelial cells (HUVECs). In contrast, we used HLECs, which may exhibit distinct regulatory mechanisms (^12,19^). We speculated that oxidative stress-induced changes in these different cell types may contribute to the modulation of *GLS2* expression, and future studies should validate these findings across different cellular models to understand their relevance in the pathogenesis of DCs. Our findings revealed that HG-treated HLECs presented considerably higher miR-195-5p levels. Moreover, the increase in miR-195-5p aggravated oxidative damage, whereas the decrease substantially alleviated oxidative impairments. Recent studies have reported that miR-195 plays a significant role in epithelial-mesenchymal transition (EMT) and fibrosis across various cell types. It may have both profibrotic and antifibrotic effects, depending on the cellular context and the specific molecular targets involved. For example, in hepatocellular carcinoma (HCC), miR-195 inhibits EMT by targeting Yes-associated protein (YAP), thereby reducing tumor metastasis (^20^). Conversely, in HG-stimulated retinal pigment epithelial cells, miR-195 was found to promote EMT by regulating VEGFA and Snail1, highlighting its context-dependent regulatory mechanisms (^21^). As EMT is a key step in fibrosis progression and oxidative stress is a key inducer of EMT via pathways such as the TGF-β/SMAD and PI3K/AKT signaling pathways, the established relationship between oxidative stress and EMT suggests that miR-195-5p may contribute to fibrotic changes in HG-induced HLECs. Although this study did not specifically assess EMT-related markers such as E-cadherin, N-cadherin, and vimentin, the role of miR-195-5p in promoting fibrosis highlights its importance in the development of DCs. Therefore, future studies should focus on evaluating EMT-associated protein and gene expression profiles to elucidate the mechanisms by which miR-195-5p drives lens epithelial fibrosis and its implications in the pathogenesis of DCs. Regarding the function of *GLS2*, previous studies focused on glioblastomas and HCCs, which act as tumor suppressors by regulating p53 (^3,22^). *GLS2* is associated with antioxidant defense, thus increasing the GSH content in cells while decreasing the ROS content (^23^). Glutamate is a vital precursor of GSH, a tripeptide containing glutamate, cysteine, and glycine; however, the relationship between *GLS2* and HG-treated HLECs is poorly understood. Our results suggested that knocking down *GLS2* promotes oxidative stress, whereas overexpressing *GLS2* decreases ROS levels and increases glutamate and GSH levels, the GSH-to-GSSG ratio, and cell viability, indicating that *GLS2* is a type of antioxidant.

*GLS2* promotes oxidative phosphorylation to generate ATP (^23^). Hence, the function of *GLS2* in aerobic metabolism was further investigated. a-Ketoglutarate is a crucial metabolite in the tricarboxylic acid cycle and plays a key role in ATP generation. Our findings revealed that *GLS2* overexpression increased the levels of a-ketoglutarate and ATP, whereas knocking down *GLS2* decreased the levels of a-ketoglutarate and ATP, indicating that *GLS2* participates in aerobic metabolism.

Agreeing with the prediction by TargetScanHuman 8.0 (https://www.targetscan.org/vert_80/), our findings showed the NC of miR-195-5p on *GLS2* mRNA. The interaction site, as well as the interaction between *GLS2* mRNA and miR-195-5p, which is a direct molecular mechanism, was demonstrated using a dual-luciferase reporter.

This study had several limitations. A rat DC lens model was not established, and lens anterior capsule specimens from DC patients were not obtained; therefore, we could not determine the link between miR-195-5p and oxidative stress in DCs. Additionally, the aerobic metabolism pathway was not thoroughly investigated in our study, particularly regarding oxygen consumption during mitochondrial respiration. While we focused on assessing ATP and α-ketoglutarate levels as key indicators of metabolic activity, these parameters provide only a partial understanding of mitochondrial function. To gain deeper insights into how miR-195-5p affects mitochondrial bioenergetics and glycolytic flux, future studies should evaluate mitochondrial respiration parameters such as the OCR and the extracellular acidification rate (ECAR). By integrating these functional assays, we aimed to comprehensively determine the regulatory effects of miR-195-5p on mitochondrial respiration and oxidative metabolism in HG-induced HLECs.

In HG-treated HLECs, miR-195-5p was found to exacerbate oxidative stress through direct negative regulation of *GLS2*, which is a vital enzyme in the glutaminolysis pathway. Besides surgical treatment, antioxidants, miR-195-5p, *GLS2*, and glutaminase might act as novel targets for treating patients with DCs.
